# Are alveolar type 2 cells “oversaturated” in obesity and ARDS?

**DOI:** 10.1152/ajplung.00325.2025

**Published:** 2025-10-14

**Authors:** Avnee J. Kumar, Mark L. Hepokoski

**Affiliations:** 1 Section of Pulmonary and Critical Care, VA San Diego Healthcare System, San Diego, California, United States; 2 Division of Pulmonary, Critical Care, Sleep Medicine, and Physiology, Department of Medicine, University of California, San Diego, California, United States

Acute respiratory distress syndrome (ARDS) is a severe form of respiratory failure with a mortality rate of up to 40% (1). The risk of ARDS is increased in patients with obesity, and the incidence of obesity is 20%–40% in the critically ill (2). Obesity-related comorbidities and complicated ventilatory mechanics are often thought to be the major mechanisms involved. However, obesity is associated with a survival benefit in some critical illnesses, which suggests that there are other physiological or molecular mechanisms that contribute to ARDS. Elucidating these mechanisms may lead to the development of novel, precision treatment approaches that could be lifesaving for numerous patients worldwide.

Preclinical studies have demonstrated that obesity leads to metabolic dysfunction, including impaired fatty acid oxidation (FAO), in the lung (3). Alveolar epithelial type 2 cells (AEC2s) are the most metabolically active cells in the lung due to the high energy demands of maintaining alveolar homeostasis, and FAO in mitochondria is critical to multiple AEC2 functions relevant to ARDS. For example, FAO in AEC2s supports the high energy requirements of active transport of ions and fluids from the alveolar space, and noncardiogenic alveolar edema is a hallmark feature of ARDS. FAO is also required for AEC2s to generate and secrete lipid-rich surfactant, and surfactant dysfunction in ARDS is a well-described contributor to alveolar collapse. Finally, FAO supports the transition of AEC2s to alveolar epithelial type 1 cells in the repair stages of ARDS.

In this journal, Kallinos et al. (4) provide a direct link between diet-induced obesity, FAO dysfunction in AEC2s, and increased lung injury due to hyperoxia (4). Specifically, they show that AEC2s from obese versus lean mice exposed to hyperoxia show increased accumulation of intracellular fatty acids, suggesting dysregulated FAO. FAO was also the most transcriptionally downregulated mitochondrial process after hyperoxia in AEC2s from obese versus lean mice. Next, they showed that these findings correlated with AEC2 metabolic dysfunction, including decreased basal respiration, maximal respiration, and ATP production. Importantly, these findings were associated with a decrease in surfactant-related phospholipids and increased bronchoalveolar lavage fluid (BALF) surface tension, consistent with impaired AEC2 function. The authors then enhanced the translational potential of these findings via a reanalysis of single-cell sequencing data from human subjects with COVID-19, which revealed a downregulation of FAO in AEC2s from those with a body mass index (BMI) of ≥30 versus <30 kg/m^2^.

This study opens the door for multiple translational opportunities targeting metabolic dysfunction to prevent ARDS in patients with obesity. For example, there are readily available therapeutics, including glucagon-like peptide-1 (GLP-1) agonists, that have demonstrated beneficial effects on fatty acid metabolism. Intriguingly, GLP-1 agonists have also been found to mitigate lung injury in a preclinical model of sepsis (5). Another study found FAO to be impaired in alveolar epithelial cells in a lipopolysaccharide (LPS) model of acute lung injury, and treatment with fenofibrate, a peroxisome proliferator-activated receptor α/peroxisome proliferator-activated receptor γ coactivator 1-α (PPAR-α/PGC-1α) activator, mitigated lung injury by restoring FAO and mitochondrial function (3). Drugs that stimulate AMP-activated protein kinase (AMPK), including metformin, are also known to restore FAO and have shown similar results in mitigating lung injury in animal models (6).

Still, multiple questions remain that limit the immediate translation of these drugs as treatments for ARDS in patients with obesity. First, the exact mechanisms of the metabolic changes identified have not been completely elucidated. In previous work, the authors demonstrated that knockout of the mitochondrial fatty acid transporter, carnitine palmitoyl transferase 1A (CPT1A), decreased chemokines in mouse models of LPS and toll-like receptor 2 agonism (7), and the present study shows increased CPT1A expression in AEC2s from obese versus lean mice. However, AEC2-specific CPT1A knockout did not impact lung injury or free fatty acid levels after hyperoxia, suggesting increased CPT1A is not deleterious in this model. The authors have also shown previously that free fatty acids accumulate in BALF after hyperoxia in diet-induced obese versus lean mice (8). They now show in an in vitro model that free fatty acids (palmitic acid) contribute directly to the metabolic derangements they describe in vivo. Still, the upstream mechanisms of free fatty acid release and the downstream pathways that contribute to metabolic function are unclear. Finally, GLP-1 agonists, PPAR-α/PGC-1α activators, and AMPK activators promote FAO in multiple cell types, but whether these effects translate to improved FAO in AEC2s remains to be seen.

It is also unclear how these findings translate to other etiologies of ARDS, particularly as the association of obesity and ARDS appears to be endotype specific. For example, there is a strong association between obesity and an increased risk of death due to ARDS induced by viral pneumonias, including influenza and COVID-19, but in some contexts, obesity is associated with improved outcomes (9, 10). Future work in other ARDS models will need to examine whether similar metabolic derangements are present, as such studies may provide new insights into the so-called “obesity paradox.” These studies are also critical to determining the optimal patients to enroll in future clinical trials. Finally, it is important to note that the present study focused exclusively on male mice, leaving open questions regarding sex differences in obesity and the metabolic derangements observed. Evaluating for differences in sex is particularly important in the context of metabolism due to mitochondria being exclusively maternally inherited.

In summary, this elegant study by Kallinos et al. (4) advances the fields of ARDS and obesity research in an impactful way. Their results reveal that diet-induced obesity leads to dysregulation in a key metabolic pathway (FAO) in an important cell type (AEC2s) involved in ARDS pathogenesis. FAO in AEC2s has the potential to be modifiable by drugs that are readily available and have an excellent safety profile. Future studies in this area should focus on deepening our understanding of the mechanisms involved to pave the way for potentially lifesaving translational studies.
